# Neonatal rotavirus vaccine (RV3-BB) immunogenicity and safety in a neonatal and infant administration schedule in Malawi: a randomised, double-blind, four-arm parallel group dose-ranging study

**DOI:** 10.1016/S1473-3099(21)00473-4

**Published:** 2022-05

**Authors:** Desiree Witte, Amanda Handley, Khuzwayo C Jere, Nada Bogandovic-Sakran, Ashley Mpakiza, Ann Turner, Daniel Pavlic, Karen Boniface, Jonathan Mandolo, Darren Suryawijaya Ong, Rhian Bonnici, Frances Justice, Naor Bar-Zeev, Miren Iturriza-Gomara, Jim Ackland, Celeste M Donato, Daniel Cowley, Graeme Barnes, Nigel A Cunliffe, Julie E Bines

**Affiliations:** a Malawi Liverpool Wellcome Trust Clinical Research Programme, Blantyre, Malawi; b Institute of Infection, Veterinary and Ecological Sciences, University of Liverpool, Liverpool, UK; c NIHR Health Protection Research Unit in Gastrointestinal Infections, University of Liverpool, Liverpool, UK; d Centre for Vaccine Innovation and Access, Program for Appropriate Technology in Health, Seattle, WA, USA; e Murdoch Children's Research Institute, Parkville, VIC, Australia; f Medicines Development for Global Health, Southbank, VIC, Australia; g Kamuzu University of Health Sciences, Blantyre, Malawi; h International Vaccine Access Center, Department of International Health, Johns Hopkins Bloomberg School of Public Health, Baltimore, MD, USA; i Global BioSolutions, Melbourne, VIC, Australia; j Department of Gastroenterology and Clinical Nutrition, Royal Children's Hospital, Parkville, VIC, Australia; k Department of Paediatrics, The University of Melbourne, Parkville, VIC, Australia

## Abstract

**Background:**

Rotavirus vaccines reduce rotavirus-related deaths and hospitalisations but are less effective in high child mortality countries. The human RV3-BB neonatal G3P[6] rotavirus vaccine administered in a neonatal schedule was efficacious in reducing severe rotavirus gastroenteritis in Indonesia but had not yet been evaluated in African infants.

**Methods:**

We did a phase 2, randomised, double-blind, parallel group dose-ranging study of three doses of oral RV3-BB rotavirus vaccine in infants in three primary health centres in Blantyre, Malawi. Healthy infants less than 6 days of age with a birthweight 2·5 to 4·0 kg were randomly assigned (1:1:1:1) into one of four treatment groups: neonatal vaccine group, which included high-titre (1·0 × 10^7^ focus-forming unit [FFU] per mL), mid-titre (3·0 × 10^6^ FFU per mL), or low-titre (1·0 × 10^6^ FFU per mL); and infant vaccine group, which included high-titre (1·0 × 10^7^ FFU per mL) using a computer generated code (block size of four), stratified by birth (singleton *vs* multiple). Neonates received their three doses at 0–5 days to 10 weeks and infants at 6–14 weeks. Investigators, participant families, and laboratory staff were masked to group allocation. Anti-rotavirus IgA seroconversion and vaccine take (IgA seroconversion and stool shedding) were evaluated. Safety was assessed in all participants who received at least one dose of vaccine or placebo. The primary outcome was the cumulative IgA seroconversion 4 weeks after three doses of RV3-BB in the neonatal schedule in the high-titre, mid-titre, and low-titre groups in the per protocol population, with its 95% CI. With the high-titre group as the active control group, we did a non-inferiority analysis of the proportion of participants with IgA seroconversion in the mid-titre and low-titre groups, using a non-inferiority margin of less than 20%. This trial is registered at ClinicalTrials.gov (NCT03483116).

**Findings:**

Between Sept 17, 2018, and Jan 27, 2020, 711 participants recruited were randomly assigned into four treatment groups (neonatal schedule high titre n=178, mid titre n=179, low titre n=175, or infant schedule high titre n=179). In the neonatal schedule, cumulative IgA seroconversion 4 weeks after three doses of RV3-BB was observed in 79 (57%) of 139 participants in the high-titre group, 80 (57%) of 141 participants in the mid-titre group, and 57 (41%) of 138 participants in the low-titre group and at 18 weeks in 100 (72%) of 139 participants in the high-titre group, 96 (67%) of 143 participants in the mid-titre group, and 86 (62%) of 138 of participants in the low-titre. No difference in cumulative IgA seroconversion 4 weeks after three doses of RV3-BB was observed between high-titre and mid-titre groups in the neonatal schedule (difference in response rate 0·001 [95%CI −0·115 to 0·117]), fulfilling the criteria for non-inferiority. In the infant schedule group 82 (59%) of 139 participants had a cumulative IgA seroconversion 4 weeks after three doses of RV3-BB at 18 weeks. Cumulative vaccine take was detected in 483 (85%) of 565 participants at 18 weeks. Three doses of RV3-BB were well tolerated with no difference in adverse events among treatment groups: 67 (39%) of 170 participants had at least one adverse event in the high titre group, 68 (40%) of 172 participants had at least one adverse event in the mid titre group, and 69 (41%) of 169 participants had at least one adverse event in the low titre group.

**Interpretation:**

RV3-BB was well tolerated and immunogenic when co-administered with Expanded Programme on Immunisation vaccines in a neonatal or infant schedule. A lower titre (mid-titre) vaccine generated similar IgA seroconversion to the high-titre vaccine presenting an opportunity to enhance manufacturing capacity and reduce costs. Neonatal administration of the RV3-BB vaccine has the potential to improve protection against rotavirus disease in children in a high-child mortality country in Africa.

**Funding:**

Bill & Melinda Gates Foundation, Australian Tropical Medicine Commercialisation Grant.

**Research in context**

**Evidence before this study**

We searched the Medline database for research done in humans published between Jan 1, 1983, and May 27, 2021, using search terms “rotavirus” and “rotavirus vaccines”.114 countries have now introduced a rotavirus vaccine into their national immunisation programme or sub-nationally. However, over 80 million or approximately 45% of children less than 5 years of age remained unvaccinated in 2019. Barriers to access and coverage are reported to include vaccine cost, gaps in supply, logistical challenges, and ongoing concerns regarding vaccine safety. Previous studies have reported that the protective efficacy provided by rotavirus vaccines is lower in low-income, high child mortality countries than in high-income, low child mortality countries. It has been proposed that maternal antibodies, the gut microbiome, environmental enteropathy, interference with oral polio vaccine, and differential expression of histo-blood group antigens within populations might be contributing factors. The first dose of the two-dose or three-dose rotavirus vaccine schedule is recommended at, or after, 6 weeks of age. Modelling studies report that a neonatal schedule for a rotavirus vaccine, with the first dose administered at birth, has the potential to prevent more rotavirus deaths and cause fewer excess intussusception deaths than the schedules currently recommended by WHO. Birth is an established Expanded Programme on Immunisation timepoint in many countries and would enable early protection from severe rotavirus disease. Asymptomatic neonatal rotavirus strains containing the P[6] VP4 genotype are structurally and functionally distinct from disease-causing rotavirus strains. Wild-type infection with the neonatal rotavirus strain RV3 (G3P[6]) was associated with protection from severe and moderate rotavirus gastroenteritis over the first 3 years of life. The human neonatal rotavirus vaccine (RV3-BB) is based on this human neonatal strain and has been shown to be well tolerated and immunogenic in a neonatal schedule with the first dose administered within 0–5 days after birth or in the routine infant schedule with the first dose from 6–8 weeks of age. Vaccine efficacy of three doses of RV3-BB vaccine (8·6 × 10^6^ focus-forming unit [FFU] per mL) administered in a neonatal schedule against severe rotavirus gastroenteritis was 94% at 12 months and 75% at 18 months of age in a high child mortality setting in Indonesia.

**Added value of this study**

This phase 2 study assessed the immunogenicity and safety of three different titres of the RV3-BB vaccine in a neonatal administration schedule in Malawian infants. This study found that three doses of oral RV3-BB were well tolerated and immunogenic when administered in an infant or neonatal schedule. A lower titre (3 × 10^6^ FFU per mL) of vaccine administered in the neonatal schedule did not have an inferior immune response (IgA seroconversion) when compared with the higher titre that has been used in previous clinical studies of the RV3-BB vaccine.

**Implications of all the available evidence**

Rotavirus vaccines are reported to reduce child death and disease due to rotavirus gastroenteritis; however, there remain challenges in providing timely access to a rotavirus vaccine for many children worldwide. The protection offered by rotavirus vaccines is reported to be lower in low-income, high child mortality countries than in high-income, low child mortality countries. A neonatal administration schedule of a rotavirus vaccine has the potential to improve the protection and safety profile provided. A vaccine based on a P[6] neonatal rotavirus strain that is known to replicate well in the newborn gut without causing symptoms is an ideal candidate to target a neonatal administration schedule. This study builds on the safety, immunogenicity, and efficacy data of RV3-BB vaccine and supports large scale manufacture of RV3-BB at a lower vaccine titre. It also shows that the human neonatal rotavirus vaccine RV3-BB is well tolerated and immunogenic when administered in a neonatal administration schedule in a high child mortality, high rotavirus disease burden country in Africa.

## Introduction

Rotavirus vaccines are cost-effective and reduce hospitalisations due to rotavirus disease worldwide.^1^ WHO recommends that all children receive a rotavirus vaccine and, to date, 114 countries have introduced a rotavirus vaccine into their national immunisation schedule. Despite this achievement over 80 million or 45% of children less than 5 years of age do not receive a rotavirus vaccine.^2^ Barriers to access and coverage of rotavirus vaccines include vaccine cost, gaps in vaccine supply, logistical challenges for timely implementation, and ongoing concerns about vaccine safety. In addition, the level of protection provided by rotavirus vaccines is lower in high child mortality countries, where rotavirus disease burden remains high and peaks early in infancy.^3^ Administration of a rotavirus vaccine from birth has the potential to address some of these challenges.^3^

Soon after the discovery of rotavirus in 1973, it was recognised that neonatal rotavirus strains containing the P[6] VP4 genotype were structurally and functionally distinct from other rotavirus strains that caused disease.^4^ Neonatal P[6] strains, such as RV3 (G3P[6]), replicate well in the newborn gut in the presence of maternal antibodies and breast milk but do not cause symptoms in infected infants.^5^ Wild-type infection with the neonatal strain RV3 was associated with protection from severe and moderate rotavirus gastroenteritis over the first 3 years of life.^6^ The RV3-BB vaccine is based on this naturally attenuated human neonatal strain (G3P[6]) and has been shown to be well tolerated and immunogenic in a neonatal schedule with the first dose administered within 0–5 days after birth or in the routine infant schedule with the first dose from 6–8 weeks of age.^7^, ^8^ Vaccine efficacy of three doses of RV3-BB vaccine (8·6 × 10^6^ focus-forming unit [FFU] per mL) administered in a neonatal schedule against severe rotavirus gastroenteritis was 94% at 12 months and 75% at 18 months of age in a high child mortality setting in Indonesia.^7^ Potential advantages of a neonatal schedule include early protection from rotavirus disease, opportunity to administer soon after birth when mother and baby are in contact with healthcare workers, and a potential for an improved safety profile as the baseline risk of intussusception is low in the first 6 weeks of life.^3^ A birth dose of an oral rotavirus vaccine may also experience fewer challenges as gastric secretions have a neutral pH, the gut microbiome is not yet matured, and the intake of breast milk is limited.^9^, ^10^

Rotavirus vaccine (Rotarix, GlaxoSmithKline) is included in Malawi's Expanded Programme on Immunisation (EPI) schedule, administered as two oral doses at 6 and 10 weeks of age. The aim of this study was to determine if the human neonatal RV3-BB vaccine administered in a neonatal schedule was immunogenic and safe in infants in Malawi and may have potential to improve rotavirus vaccine impact in African children. The study also aimed to compare anti-rotavirus IgA seroconversion using a lower titre of RV3-BB (3·0 × 10^6^, 1·0 × 10^6^ FFU per mL) compared with the higher titre (approximately 1 × 10^7^ FFU per mL) used in the previous clinical trials, to inform future large-scale manufacture of RV3-BB.

## Methods

### Study design and participants

This was a phase 2, randomised, double-blind, placebo masked allocation, four-arm parallel group, dose-ranging study of oral human neonatal rotavirus vaccine (RV3-BB) administered at a titre of 1·0 × 10^7^, 3·0 × 10^6^, or 1·0 × 10^6^ as a three-dose neonate schedule, or administered at a titre of 1·0 × 10^7^ as a three-dose infant schedule. The study was done in three health centres in the Blantyre district (Ndirande, Bangwe, and Limbe) and included participants recruited from the Queen Elizabeth Central Hospital in Blantyre, Malawi. Healthy babies less than 6 days (or 144 hours) of age with a birth weight of 2·5 to 4·0 kg, irrespective of in-utero exposure to human immunodeficiency virus, were eligible for randomisation. Preliminary written informed consent was obtained from pregnant women before delivery. Final written informed consent was obtained following birth before confirming eligibility. The protocol was approved by the National Health Science Research Committee Malawi, the Ethics committees of the Royal Children's Hospital Melbourne and the University of Liverpool, and the Malawi Pharmacy Medicines and Poisons Board. The study was done in accordance with International Council for Harmonisation Good Clinical Practice guidelines. The study sponsor was Murdoch Children's Research Institute. An independent data safety monitoring board regularly reviewed safety data. Study conduct was monitored by an independent contract research organisation (TCD Global, South Africa); data management and statistical analysis was conducted by TCD Global and an independent statistical consultant (WR).

### Randomisation and masking

Eligible infants were randomly assigned into one of four treatment arms (neonatal vaccine group: high-titre group [1·0 × 10^7^], mid-titre group [3·0 × 10^6^], or low-titre group [1·0 × 10^6^] or infant vaccine group high-titre group [1·0 × 10^7^]) in a 1:1:1:1 ratio. The randomisation code was generated by the randomisation statistician (TCD Global) with block randomisation (block size of four), stratified by birth (singleton *vs* multiple) using SAS software (version 9.4). Multiple births had separate randomisation numbers but randomised to the same treatment arm. The randomisation code was assigned to each participant in order of enrolment. Intervention product (RV3-BB or placebo) doses were drawn into syringes for dispensing by an unmasked pharmacist who was not involved in other aspects of the study conduct or data analysis at the central pharmacy in the Malawi Liverpool Wellcome Trust Clinical Research Programme, Blantyre. The RV3-BB vaccine and placebo were indistinguishable in appearance and participating families, investigators, and laboratory staff were masked to treatment allocation.

### Procedures

Clinical trial lots were prepared at Meridian Life Sciences (Memphis, TN, USA) to a titre of 1·0 × 10^7^ (high titre), 3·0 × 10^6^ (mid titre), and 1·0 × 10^6^ (low titre) FFU per mL in serum free media supplemented with 10% sucrose. Placebo contained the same media with 10% sucrose. Intervention product vials were stored at −70°C until thawed within 6 h before administration.

**Figure 1:**
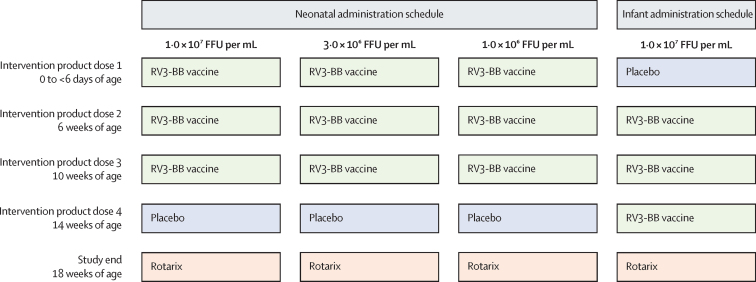
Study design FFU=focus forming unit. RV3-BB=human neonatal rotavirus vaccine.

Participants received four 1 mL oral doses of intervention product according to their allocated treatment group, with doses administered at 0–5 days (intervention product dose 1), 6 weeks (intervention product dose 2), 10 weeks (intervention product dose 3), and 14 weeks of age (intervention product dose 4; figure 1). The four doses of intervention product consisted of three doses of 1 mL RV3-BB vaccine and one 1 mL dose of placebo. Intervention product doses two, three, and four were preceded by a 2 mL dose of antacid solution (Mylanta Original, Broadway, NSW, Australia). No placebo only group was used as a rotavirus vaccine was included in the EPI schedule in Malawi. The intervention product was co-administered with routine vaccines in the Malawian National EPI schedule with the exception for Rotarix. As it had been planned for the inactivated polio vaccine (IPV) to be introduced into the Malawi EPI schedule during this study, all study participants were administered IPV instead of oral polio vaccine. Participants were followed until 18 weeks of age with a minimum of monthly face-to-face visits. At completion of all study visits and procedures (18 weeks of age), a single 1·5 mL dose of Rotarix vaccine was administered to ensure that participants were protected from severe rotavirus disease in the event that the study vaccine was not protective. Clinical data were reported using web based electronic data capture (Nukleus version 1.4).

### Outcomes

The primary outcome was to assess anti-rotavirus IgA seroconversion (henceforth called IgA seroconversion, which was defined as a ≥three-times increase from baseline) 4 weeks after three doses of RV3-BB administered in a neonatal schedule at a vaccine titre of 1·0 × 10^7^, 3·0 × 10^6^, or 1·0 × 10^6^ FFU per mL. A secondary outcome of the study was IgA seroconversion after three doses of RV3-BB 1·0 × 10^7^ FFU per mL administered in the infant schedule. Blood was collected from the cord at delivery, and venous blood was collected immediately before intervention product dose two, three, and four, and 28 days after intervention product dose four. Baseline (prevaccine) was defined as cord blood (or venous blood taken at birth if cord blood not available) for the neonatal schedule comparison or blood drawn before intervention product dose two for the infant schedule comparison. Serum rotavirus IgA antibody titres were measured by ELISA using rabbit anti-RV3 polyclonal antisera as the coating antibody and RV3-BB virus or Vero cell lysate as the capture antigen.^8^ The geometric mean titre of the serum IgA response after three doses of RV3-BB was measured.

Vaccine virus-like shedding was determined in a stool sample collected on days 3 to 5 after each intervention product dose using a rotavirus VP6 specific reverse transcription PCR assay with VP6 positive amplicons confirmed by sequence analysis.^8^ Positive vaccine take was defined as IgA seroconversion or stool shedding after intervention product administration. Cumulative vaccine take was defined as positive vaccine take after intervention product dose one, two, or three for the neonatal schedule, and after intervention product doses two, three, or four for the infant schedule.

Vital signs were assessed at each study visit and participants were observed for 30 min after intervention product administration. All adverse events occurring up to 28 days after administration of intervention product doses were recorded. Serious adverse events were defined as any untoward health occurrence that resulted in death, hospitalisation, or were considered to be medically significant or life threatening occurring up to 28 days after the last dose of intervention product. Causality and severity grading of adverse events were determined by the investigator. Specific events such as diarrhoea and episodes of blood-in-stool were recorded.

All episodes of gastroenteritis during the study period were clinically assessed and stool samples collected. Gastroenteritis was defined as three or more stools looser than normal for that child within a 24-h period presenting for care or actively ascertained by the study team if at home. Severe gastroenteritis was defined as gastroenteritis with a modified Vesikari score of 11 or higher.^11^ Rotavirus gastroenteritis was defined as an episode of gastroenteritis with rotavirus antigen detected in the stool by commercial ELISA (Premier Rotaclone EIA kit Meridian Bioscience, Cincinatti, OH, USA). Positive samples were genotyped by heminested multiplex reverse transcription PCR.^8^

### Statistical analysis

Data were analysed in four participant populations. The full-analysis set included all participants randomised into the study, the safety-analysis population consisted of all participants who received at least one dose of intervention product, and the intention-to-treat analysis population included all participants that received at least one dose of RV3-BB and had at least one evaluable serum sample after a dose of intervention product. The primary analysis was based on the per-protocol population, including only participants who completed the study in compliance with the protocol and who reported no major protocol violations that might effect on the primary endpoint, with secondary analysis conducted in the intention-to-treat population.

With the high-titre (1·0 × 10^7^ FFU per mL) RV3-BB neonatal schedule as the active control group, the sample size was calculated to show the non-inferiority of the lower titre (3·0 × 10^6^ and 1·0 × 10^6^ FFU per mL) vaccine group with respect to the proportion of participants who have an IgA seroconversion 4 weeks after three doses of RV3-BB. We estimated that 30% of participants would be excluded from the per-potocol population due to death, study withdrawal, loss to follow-up, or study non-compliance and a 50% IgA seroconversion probability was assumed for the active controls. Based on a one-sided 0·025 level score test with 90% power under the alternative of no difference in response probabilities, 172 participants per group were required for a total sample size of 688 participants.

A non-inferiority analysis of the mid-titre and low-titre groups was compared with the high-titre group with the difference in proportions in the IgA seroconversion rate and its 95% CI calculated. Non-inferiority of the lower titre groups was shown if the upper bound of the CI was below 20%. The 95% CI of the difference in the IgA seroconversion rate between vaccine titre groups was also calculated using the method of Newcombe-Wilson, without continuity correction for the difference between binomial proportions as implemented in PROC FREQ (SAS version 9.4). Measurements below the lower limit of detection were assigned a value of half of limit for analysis (eg, if anti-rotavirus serum IgA is <20 [including zero], this was assigned a value of ten). As maternal serum IgA is acknowledged not to cross the placenta, participants with missing baseline cord blood IgA measurement were assigned a value below the lower limit of detection (ie, a value of ten) for the calculation of IgA seroconversion although the cord blood IgA value was acknowledged as missing when summarising titre. A sensitivity analysis was done with all available cord blood results to ensure there was no bias in reporting. Anti-rotavirus serum IgA titres were also summarised at each serum collection timepoint, by treatment group using geometric means and the mean and standard deviation on the log scale.

Difference in responses rates and its 95% CI were based on Pearson's χ^2^ test for IgA seroconversion, stool shedding and vaccine take at timepoint, and for the difference in response rates between treatment groups. For the analysis of vaccine take a participant was defined as missing only if all components of the outcome were missing. Safety data were summarised by group using the safety-analysis population. This trial was registered with Clinicaltrials.gov and is now closed to recruitment (NCT03483116).

### Role of the funding source

The funder of the study had a limited role in initial discussions of the study design, but no role in study protocol development, data collection or analysis, data interpretation or the writing of this report.

## Results

**Figure 2:**
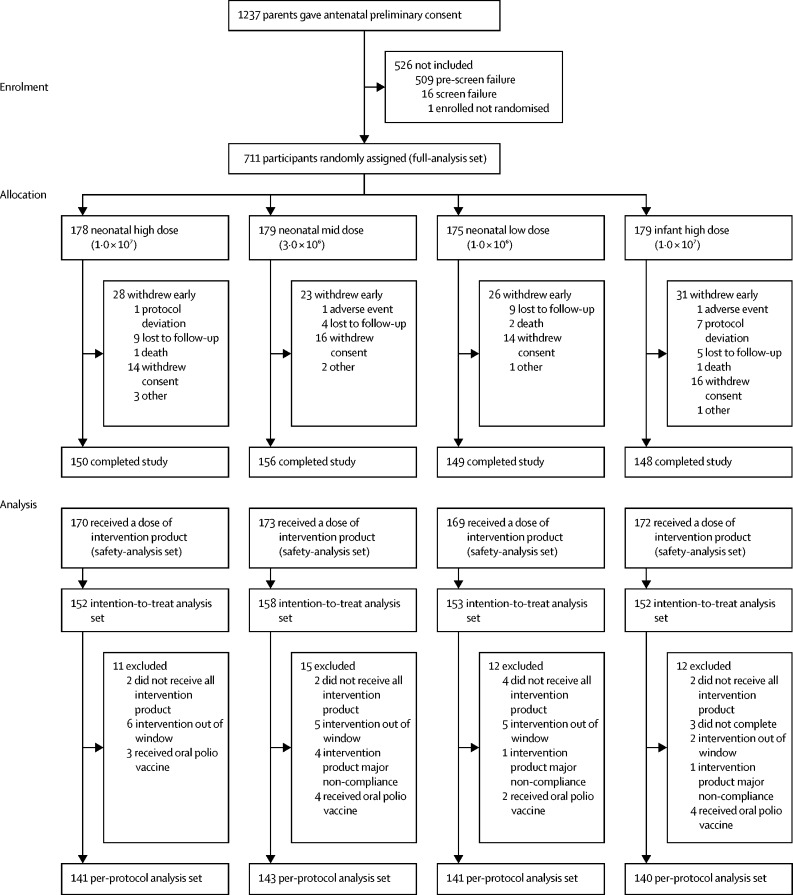
Randomisation, trial assigment, and follow-up

Between Sept 17, 2018, and Jan 27, 2020, 711 participants were randomised into four treatment groups (neonatal schedule high titre n=178, mid titre n=179, low titre n=175; or infant schedule high titre n=179; figure 2). There were no significant differences in baseline demographic characteristics between groups (table 1). 684 (96%) participants received at least one dose of intervention product (safety-analysis population) and 603 (85%) participants were followed-up to final visit at 18 weeks of age (figure 2). At least one evaluable blood sample for assessment of serum IgA was available from 615 (87%) participants (intention-to-treat population). The per-protocol population included 565 (80%) participants who completed the study in compliance with the protocol with no significant protocol violations (figure 2). The mean age at administration of the first dose of intervention product was 1·5 days (SD 1·43 days).

**Table 1:** Participant disposition

		**Safety population**				**Per-protocol population**			
		High-titre neonatal (1 × 10^7^ FFU per mL)	Mid-titre neonatal (3 × 10^6^ FFU per mL)	Low-titre neonatal (1 × 10^6^ FFU per mL)	High-titre infant (1 × 10^7^ FFU per mL)	High-titre neonatal (1 × 10^7^ FFU per mL)	Mid-titre neonatal (3 × 10^6^ FFU per mL)	Low-titre neonatal (1 × 10^6^ FFU per mL)	High-titre infant (1 × 10^7^ FFU per mL)
Sex									
	Male	97 (57%)	80 (46%)	85 (50%)	92 (53%)	82 (58%)	68 (48%)	70 (50%)	75 (54%)
	Female	73 (43%)	93 (54%)	84 (50%)	80 (47%)	59 (42%)	75 (52%)	71 (50%)	65 (46%)
Black African ethnicity		170 (100%)	173 (100%)	169 (100%)	172 (100%)	141 (100%)	143 (100%)	141 (100%)	140 (100%)
Gestational age, weeks		37·6 (1·21)	37·3 (1·02)	37·5 (1·08)	37·6 (1·25)	37·6 (1·16)	37·3 (1·0)	37·5 (1·06)	37·6 (1·26)
Birthweight, g		3106 (343·4)	3100·5 (378·0)	3120·2 (351·7)	3161·8 (365·1)	3089·4 (344·5)	3117·9 (384·4)	3121·9 (359·6)	3150·8 (362·6)
Age at first dose of intervention product, days		1·5 (1·5)	1·4 (1·42)	1·4 (1·45)	1·5 (1·35)	1·5 (1·49)	1·4 (1·37)	1·5 (1·45)	1·7 (1·43)

Data are n (%) or mean (SD). FFU=focus forming units.

**Figure 3:**
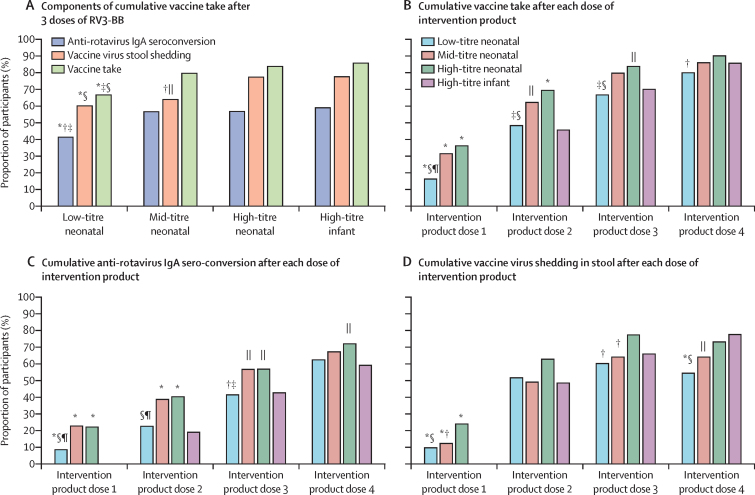
Vaccine response across treatment groups RV3-BB=human neonatal rotavirus vaccine. *p<0·005 compared with infant high-titre. †p<0·05 compared with neonatal high-titre. ‡p<0·05 compared with neonatal mid-titre. §p<0·005 compared with neonatal high-titre. ¶p<0·005 compared with neonatal mid-titre. ||p<0·05 compared with infant high-titre.

Cumulative IgA seroconversion 4 weeks after administration of three doses of RV3-BB in the neonatal schedule was observed in 79 (57%) of 139 participant in the high titre group, 80 (57%) of 141 participant in the mid titre group, and 57 (41%) of 138 of participants in the low-titre groups (table 2). No difference was observed in cumulative IgA seroconversion after three RV3-BB doses between the high-titre and mid-titre groups administered in the neonatal schedule (difference in response rate 0·001 [95% CI −0·115 to 0·117]) with upper and lower bounds of the confidence intervals less than 20%, fulfilling criteria for non-inferiority. There was no difference in cumulative vaccine take after three doses in the mid-titre group and high-titre group in the neonatal schedule (figure 3 A, B). In the low-titre group a lower proportion of participants had cumulative IgA seroconversion detected 4 weeks after administration of three doses of RV3-BB than in the high-titre and mid-titre groups (difference in proportions: high-titre *vs* low-titre 0·155 [95% CI 0·039 to 0·272]; mid-titre *vs* low-titre 0·154 [95% CI 0·038 to 0·270]). However, no difference was observed at 18 weeks of age when all participants had received three vaccine doses in the neonatal schedule groups (high-titre 100 (72%) of 139; mid-titre 96 (67%) of 143; low-titre 86 (62%) of 138; table 2; figure 3C). Stool vaccine-like shedding was detected in 218 (67%) of 324 participants after a dose of RV3-BB in the neonatal schedule groups (table 2). Cumulative vaccine take was detected in 326 (77%) of 425 participants in the neonatal schedule groups assessed 4 weeks after three doses of RV3-BB (table 2). At 18 weeks, cumulative vaccine take was detected in 363 (85%) of 425 participants in the neonatal schedule groups, with no difference between the treatment groups (table 2). After one dose of RV3-BB in the neonatal schedule, the proportion of participants with IgA seroconversion was higher across all titre groups than in the infant schedule group that received placebo at intervention dose one (high titre 31 [22%] of 140, mid-titre 32 [23%] of 141, low-titre 12 [9%] of 141 *vs* infant placebo dose 0 [0%]; figure 3C). After one dose of vaccine in the neonatal schedule, stool shedding was observed in 29 (24%) of 121 participants in the high-titre group, 15 (12%) of 121 in the mid-titre group, and 12 (10%) of 124 in the low-titre group and vaccine take was observed in 51 (36%) of 141 in the high-titre group and 45 (31%) of 143 in the mid-titre group (figure 3 B, D). After two doses of RV3-BB cumulative IgA seroconversion was observed in 56 (40%) of 139 participants in the high-titre neonatal group and 55 (39%) of 142 participants mid-titre neonatal groups, respectively (figure 3C). Cumulative vaccine take was detected in 98 (70%) of 141 in the high-titre and 89 (62%) of 143 in the mid-titre neonatal group (figure 3B).

**Table 2:** Cumulative anti-rotavirus IgA seroconversion, vaccine virus shedding and vaccine take across vaccine treatment groups (per-protocol population)

	**High-titre neonatal (1 × 10^7^ FFU per mL)**	**Mid-titre neonatal (3 × 10^6^ FFU per mL)**	**Low-titre neonatal (1 × 10^6^ FFU per mL)**	**High-titre infant (1 × 10^7^ FFU per mL)**	**Total (N=565)**
4 weeks after RV3-BB dose three*					
Anti-rotavirus IgA seroconversion	79/139 (57%)	80/141 (57%)	57/138 (41%)	82/139 (59%)	298/557 (54%)
Vaccine virus shedding	85/110 (77%)	71/111 (64%)	62/103 (60%)	90/116 (78%)	308/440 (70%)
Vaccine take	118/141 (84%)	114/143 (80%)	94/141 (67%)	120/140 (86%)	446/565 (79%)
Anti-rotavirus serum IgA geometric mean titre	48·4 (n=139)	39·9 (n=141)	28·0 (n=135)	77·7 (n=136)	45·3 (n=551)
**4 weeks after intervention product dose four**†					
Anti-rotavirus IgA seroconversion	100/139 (72%)	96/143 (67%)	86/138 (62%)	82/139 (59%)	364/559 (65%)
Vaccine virus shedding	87/119 (73%)	71/111 (64%)	62/114 (54%)	90/116 (78%)	310/460 (67%)
Vaccine take	127/141 (90%)	123/143 (86%)	113/141 (80%)	120/140 (86%)	483/565 (85%)
Anti-rotavirus serum IgA geometric mean titre	51·6 (n=138)	59·1 (n=141)	40·1 (n=136)	77·7 (n=136)	55·6 (n=551)

RV3-BB=human neonatal rotavirus vaccine.

* For neonatal schedule vaccine from 28 days after dose 1 (0–5 days of age) to 28 days after dose 3 (approximately 14 weeks of age); for infant schedule vaccine from 28 days after dose 1 (approximately 6 weeks of age) to 28 days after dose 3 (approximately 18 weeks of age).

† All groups assessed at the same age timepoint at 18 weeks of age aligning with post-dose three in the infant schedule.

Cumulative IgA seroconversion was observed in 82 (59%) of 139 participants 4 weeks after receiving three doses of RV3-BB at high-titre in the infant schedule. When three doses of the same titre (high-titre) were administered in the neonatal or infant schedule, cumulative IgA seroconversion was higher in the neonatal schedule group at 18 weeks (difference in proportions: 0·130 [95% CI 0·018 to 0·237]) but not at 4 weeks after the third vaccine dose (difference in response rate: −0·022 [–0·138 to 0·094]; table 2). After three doses of RV3-BB in the infant schedule, 90 (78%) of 116 participants had cumulative stool shedding and vaccine take was observed in 120 (86%) of 140 participants (table 2). 26 (19%) of 137 participants had IgA seroconversion after one dose of RV3-BB in the infant schedule, which was similar to that observed in the low-titre group (difference in response rate: −0·105 [–0·185 to −0·024]) but lower than in the mid-titre (difference in response rate: 0·037 [–0·058 to 0·133]) and high-titre neonatal group (difference in response rate: 0·032 [–0·063 to 0·127]).

To account for the difference in age at receipt of RV3-BB vaccine, the safety assessment is presented according to all intervention product doses for each treatment group (intervention product doses one to four) and according to RV3-BB vaccine dose (neonatal schedule intervention product doses one to three, infant schedule intervention product doses two to four; table 3; appendix p 1). Three doses of the RV3-BB vaccine were well tolerated with no significant difference in the number of total adverse events or serious adverse events between treatment groups (table 3). Only one serious adverse event was assessed by the investigator as possibly related to intervention product. This serious adverse event was an episode of vomiting that occurred after the first dose of intervention product (placebo) in the infant schedule group. There were four fatal unrelated events from randomisation until final visit at 18 weeks of age: one in the high-titre group and two in the low-titre neonatal group and one in the infant group (appendix p 4). Two adverse events resulted in early withdrawal from the study due to a diagnosis of congenital heart disease after receipt of the first intervention product dose and another due to constipation. No episodes of blood in the stool or intussusception were reported. A total of 73 episodes of diarrhoea were reported with rotavirus detected in nine (14%) of 65 episodes with stools available for testing (appendix p 5). In the five episodes clinically assessed as severe, rotavirus was detected in the stool in three; one participant in the mid-titre group (G12P[6]) and two participants in the low-titre group (G12P[6], G9P[4]).

**Table 3:** Summary of adverse events

	**RV3-BB neonate 1 × 10^7^ FFU/mL (n=170)**	**RV3-BB neonate 3 × 10^6^ FFU/mL (n=172)**	**RV3-BB neonate 1 × 10^6^ FFU/mL (n=169)**	**RV3-BB infant 1 × 10^7^ FFU/mL (n=173)**	**Total (n=684)**
**Number of participants and events**					
Adverse events after any dose of intervention product	67 (39%); 124	68 (40%); 134	69 (41%); 119	60 (35%); 91	264 (39%); 468
Serious adverse events after any dose of intervention product	11 (7%); 12	7 (4%); 7	8 (5%); 9	5 (3%); 6	31 (5%); 34
Serious adverse events after a dose of RV3-BB*	9 (5%); 9	6 (4%); 6	7 (4%); 7	2 (1%); 2	24 (4%); 25
Grade 3 or 4 adverse events	5 (3%); 5	4 (2%); 4	5 (3%); 5	1 (<1%); 1	15 (2%); 15
Adverse events leading to death	1 (1%); 1	0	2 (1%); 2	1 (<1%); 1	4 (1%); 4
Adverse events leading to early withdrawal	0	1 (1%); 1	0	0	1 (<1%); 1
**Adverse events relatedness to intervention product**					
Related	6 (4%); 7	7 (4%); 8	1 (1%); 1	3 (2%); 4	17 (3%); 20
Not related	67 (39%); 117	64 (37%); 126	69 (41%); 118	60 (35%); 87	260 (38%); 448
Intervention product related serious adverse events	0	0	0	1 (<1%); 1*†	1 (<1%); 1

Data are number of participants (%); number of events. RV3-BB=human neonatal rotavirus vaccine.

* Age dissociated: neonatal schedule vaccine dose one at birth to dose three at 14 weeks; infant schedule vaccine dose one at 6 weeks and dose three at 18 weeks.

† After placebo at dose one.

## Discussion

RV3-BB vaccine was well tolerated and immunogenic when administered in a neonatal or infant schedule in Malawian infants. This study builds on data from a phase 2b trial in Indonesian infants, where three doses of the RV3-BB vaccine administered in the neonatal schedule were associated with a 94% protective efficacy against severe rotavirus disease at 12 months and 75% at 18 months of age.^7^ These results support an alternative approach to oral rotavirus vaccination, employing the human neonatal rotavirus vaccine RV3-BB (G3P[6]) with the first dose administered at birth.

In this study cumulative serum IgA seroconversion was observed in 216 (52%) of 418 participants 4 weeks after administration of three doses of RV3-BB administered in the neonatal schedule. As there was no placebo used as an analytical control in this study, IgA seroconversion was also assessed in the neonatal schedule group compared to the infant schedule group at 18 weeks of age, to account for any background exposure to wild-type rotavirus during the intervening 4-week period between intervention product dose three and intervention product dose four. At 18 weeks cumulative IgA seroconversion was detected in 282 (67%) of 420 participants in the neonatal schedule group compared with 82 (59%) of 139 participants in the infant schedule group. Of note, only one participant (low-titre group) with no previous evidence of vaccine take after three doses had an episode of rotavirus positive diarrhoea between intervention product doses three and four to account for seroconversion at week 18. These results compare favourably with data from current WHO prequalified rotavirus vaccines implemented across Africa.^12^, ^13^, ^14^ In the pivotal phase 3 trial in Malawi, two doses of Rotarix were associated with IgA seroconversion of 47% (95% CI 30–64) and three doses associated with a seroconversion rate of 57% (42–72).^14^ The Rotarix vaccine is currently administered in a two-dose schedule at age 6 and 10 weeks in the Malawi EPI schedule and has been associated with a vaccine effectiveness of 64% against hospitalisation due to severe rotavirus disease.^15^

The first line of protection from rotavirus infection is provided by secretory IgA on the gut mucosa; therefore, it is likely that serum anti-rotavirus IgA underestimates the level of protection provided by a rotavirus vaccine.^16^, ^17^ This presents an additional challenge for the assessment of the immune response after a birth dose of a rotavirus vaccine, as IgA does not cross the placenta and serum IgA responses might not be as reliable in the first weeks of life.^18^ As a further measure of vaccine response, we assessed shedding of vaccine virus in stool 3 to 5 days after vaccine administration, to capture the active replication of the vaccine virus. Additionally, vaccine take was assessed using a combination of IgA seroconversion and stool vaccine virus shedding. Cumulative vaccine take was detected in over two-thirds (67%) of participants assessed 4 weeks after three doses of RV3-BB administered in either the neonatal or infant schedule and between 80% to 90% at the 18 weeks of age timepoint and is consistent with previous clinical trials of RV3-BB.^7^, ^8^

There are potential advantages for a neonatal administration schedule for a rotavirus vaccine. A dose of an oral rotavirus vaccine at birth provides the earliest opportunity to stimulate the developing mucosal immune system to protect against subsequent exposure to wild-type rotavirus. Furthermore, use of an asymptomatic human neonatal rotavirus strain that binds to specific receptors in the neonatal gut avoids the potential risk of vaccine-associated diarrhoea or liver dysfunction.^19^, ^20^ In this study when RV3-BB was administered at the same titre in the neonatal and infant schedule, a higher proportion of participants in the neonatal schedule had cumulative IgA seroconversion after three doses at 18 weeks of age (difference in proportions 0·130 [95% CI 0·018–0·237]) In a head to head modelling analysis comparing efficacy and waning over time, the neonatal schedule appeared to offer more durable protection.^21^ As naturally occurring intussusception is rare in the first weeks of life, a first dose of a rotavirus vaccine delivered at birth could improve the safety profile.^3^

As RV3-BB contains the P[6] genotype, it may have intrinsic advantages for protection in infants in Africa and Asia. It has been postulated that global variations in rotavirus genotypes may, in part, be explained by differences in histo-blood group antigen status, specifically Lewis and secretor status, within the population.^22^, ^23^ Histo-blood group antigens function as a rotavirus receptor on the gut epithelium. Rotavirus strains and vaccines containing a P[8] genotype seem only able to infect Lewis- and secretor-positive individuals, whereas P[6] rotavirus strains and the RV3-BB vaccine containing the P[6] genotype are able to infect both secreter-positive and negative individuals.^22^, ^23^, ^24^ Approximately 75–80% of the population of North America, Europe and central Asia are secretors whereas the prevalence of Lewis-negative phenotype is between 20–35% in some African countries.^23^, ^24^, ^25^ This may in part explain why P[6] strains are an important cause of rotavirus disease in Africa, and why rotavirus vaccines based on the P[8] genotype are not as effective in offering protection to African infants compared with infants in Europe, the US and other high income settings.^22^, ^25^

This study assessed the impact of lower titres of vaccine virus on anti-rotavirus IgA sero-responses in to inform plans for future large-scale vaccine manufacture. IgA seroconversion 4 weeks after three doses of RV3-BB was similar in the mid- and high-titre groups supporting the manufacture of the mid-titre RV3-BB vaccine. Lowering the titre of the vaccine would reduce the cost of goods and improve manufacturing efficiency and produce RV3-BB vaccine at a cost of less than US$5 for a three dose course of a 2–8°C formulated vaccine.^26^ This meets the 2020 UNICEF tender price of US$0·85–3·20 per dose or $2·55–9·60 per course.^27^ The BioFarma Indonesia manufactured high-titre RV3 vaccine is currently in a phase 3 clinical trial (NCT04185545) with plans to introduce into the Indonesian National Immunisation Program in 2023. At the time when the decision on the dose to use in the phase 3 trial was made, the results of the trial reported here were not yet available. Future vaccine development can now consider a lower vaccine titre with development of a 2–8°C formulation.^26^

The absence of statistical power to assess protective efficacy for the RV3-BB vaccine in Malawian infants is a limitation of this study, particularly as anti-rotavirus IgA seroconversion is an imperfect serological correlate of protection and newborns might not mount a robust serum IgA response. As a rotavirus vaccine (Rotarix) is currently implemented in the EPI program in Malawi a placebo group could not be included to evaluate background serum immune responses. However, the placebo was administered at intervention product dose one in the infant schedule group, provided an opportunity to assess serum IgA responses in the absence of RV3-BB in the first 6 weeks of life. Participants were enrolled irrespective of their HIV exposure status and anti-HIV prophylaxis or treatment which might have modified serum IgA responses although this has not been observed in other studies of live oral rotavirus vaccines.^28^, ^29^, ^30^

RV3-BB was observed to be safe with robust immune responses and evidence of vaccine take in infants in Malawi in a neonatal administration schedule and in an infant administration schedule. The mid-titre group had similar immune responses and vaccine take to the high-titre group although a lower immune response was observed in the low-titre group. This supports large scale manufacture at mid titre with the aim to improve manufacturing capacity and reduce costs. The neonatal administration schedule takes advantage of the intrinsic characteristics of the RV3-BB vaccine and has the potential to improve protection against rotavirus disease in children in Africa.

## Data sharing

Deidentified group data may be available for sharing on application to the corresponding author. This application must include the relevant proposal detailing the intended use of the data, the ethics approval for this proposal and requires a signed data sharing agreement. Additional study documents including the study protocol, statistical analysis plan and informed consent are available on application to the corresponding author on publication of this report**.**

## Declaration of interests

JEB is the lead of the Enteric Diseases group at MCRI (MCRI holds the license for RV3-BB vaccine). JEB is the Director of the Australian Rotavirus Surveillance Program funded by the Australian Government Department of Health and GlaxoSmithKline. MCRI and GLB hold the patent for the RV3-BB vaccine. NAC is affiliated to the National for Health Research Health Protection Research Unit in Gastrointestinal Infections at University of Liverpool, in partnership with Public Health England, in collaboration with University of Warwick. NAC is based at The University of Liverpool. The views expressed are those of the authors and not necessarily those of the National Institute of Health Research, the Department of Health and Social Care, or Public Health England. CMD has served on advisory boards for GSK (between 2019 and 2021) with all payments directed to an administrative fund held by MCRI.
