# Supplementary material for: Neonatal rotavirus vaccine (RV3-BB) immunogenicity and safety in a neonatal and infant administration schedule in Malawi: a randomised, double-blind, four-arm parallel group dose-ranging study
Source: Lancet Infect Dis. 2022 May;22(5):668–78. doi: 10.1016/S1473-3099(21)00473-4 (PMC9021029; doi:10.1016/S1473-3099(21)00473-4)
Supplement: Supplementary appendix [file mmc1.pdf]

THE LANCET
Infectious Diseases

Supplementary appendix

This appendix formed part of the original submission and has been peer reviewed. We post it as supplied by the authors.

Supplement to: Witte D, Handley A, Jere KC, et al. Neonatal rotavirus vaccine (RV3-BB) immunogenicity and safety in a neonatal and infant administration schedule in Malawi: a randomised, double-blind, four-arm parallel group dose-ranging study. *Lancet Infect Dis* 2022; published online Jan 20. https://doi.org/10.1016/S1473-3099(21)00473-4.

Supplement Table 1 Unsolicited AE by Dose and overall

MedDRA System Organ Class	RV3-BB 1.0 × 10^7 FFU/mL (Neonatal) (N=170) n (%) m	RV3-BB 3.0 × 10^6 FFU/mL (Neonatal) (N=172) n (%) m	RV3-BB 1.0 × 10^6 FFU/mL (Neonatal) (N=169) n (%) m	RV3-BB 1.0 × 10^7 FFU/mL (Infant) (N=173) n (%) m
**All IP Doses Any AEs**	**67 (39) 124**	**68 (40) 134**	**69 (41) 119**	**60 (35) 91**
Congenital, familial and genetic disorders	1 (1) 1	0 (0) 0	0 (0) 0	0 (0) 0
Eye disorders	0 (0) 0	0 (0) 0	0 (0) 0	1 (1) 1
Gastrointestinal disorders	11 (7) 14	14 (8) 21	11 (7) 14	8 (5) 10
General disorders and administration site conditions	7 (4) 7	8 (5) 8	5 (3) 5	5 (3) 6
Infections and infestations	54 (32) 79	57 (33) 87	56 (33) 79	49 (28) 62
Injury, poisoning and procedural complications	3 (2) 3	0 (0) 0	1 (1) 1	1 (1) 1
Neoplasms benign, malignant and unspecified (incl cysts and polyps)	0 (0) 0	1 (1) 1	0 (0) 0	0 (0) 0
Nervous system disorders	0 (0) 0	1 (1) 1	0 (0) 0	0 (0) 0
Pregnancy, puerperium and perinatal conditions	3 (2) 3	0 (0) 0	0 (0) 0	0 (0) 0
Reproductive system and breast disorders	1 (1) 1	0 (0) 0	0 (0) 0	0 (0) 0
Respiratory, thoracic and mediastinal disorders	6 (4) 7	6 (4) 8	9 (5) 9	3 (2) 4
Skin and subcutaneous tissue disorders	8 (5) 9	7 (4) 8	10 (6) 11	7 (4) 7
**IP Dose One Number of participants received IP Dose One**	**170**	**172**	**169**	**173**
**Any AEs**	**34 (20) 48**	**33 (19) 45**	**24 (14) 31**	**22 (13) 30**
Congenital, familial and genetic disorders	1 (1) 1	0 (0) 0	0 (0) 0	0 (0) 0
Eye disorders	0 (0) 0	0 (0) 0	0 (0) 0	1 (1) 1
Gastrointestinal disorders	4 (2) 5	5 (3) 7	4 (2) 5	3 (2) 3
General disorders and administration site conditions	5 (3) 5	3 (2) 3	1 (1) 1	2 (1) 2
Infections and infestations	22 (13) 25	27 (16) 31	19 (11) 21	15 (9) 20
Injury, poisoning and procedural complications	1 (1) 1	0 (0) 0	0 (0) 0	0 (0) 0

MedDRA System Organ Class	RV3-BB 1.0 × 10^7 FFU/mL (Neonatal) (N=170) n (%) m	RV3-BB 3.0 × 10^6 FFU/mL (Neonatal) (N=172) n (%) m	RV3-BB 1.0 × 10^6 FFU/mL (Neonatal) (N=169) n (%) m	RV3-BB 1.0 × 10^7 FFU/mL (Infant) (N=173) n (%) m
Pregnancy, puerperium and perinatal conditions	3 (2) 3	0 (0) 0	0 (0) 0	0 (0) 0
Respiratory, thoracic and mediastinal disorders	2 (1) 2	0 (0) 0	0 (0) 0	1 (1) 1
Skin and subcutaneous tissue disorders	5 (3) 5	3 (2) 4	4 (2) 4	3 (2) 3
**IP Dose Two: Number of participants received IP Dose Two**	**158**	**159**	**157**	**161**
**Any AEs**	**18 (11) 23**	**24 (15) 36**	**26 (17) 33**	**14 (9) 15**
Gastrointestinal disorders	2 (1) 2	4 (3) 5	4 (3) 5	2 (1) 2
General disorders and administration site conditions	1 (1) 1	3 (2) 3	2 (1) 2	1 (1) 1
Infections and infestations	13 (8) 15	18 (11) 22	17 (11) 19	10 (6) 10
Injury, poisoning and procedural complications	2 (1) 2	0 (0) 0	1 (1) 1	1 (1) 1
Neoplasms benign,malignant and unspecified (incl cysts and polyps)	0 (0) 0	1 (1) 1	0 (0) 0	0 (0) 0
Nervous system disorders	0 (0) 0	1 (1) 1	0 (0) 0	0 (0) 0
Respiratory, thoracic and mediastinal disorders	1 (1) 2	3 (2) 4	4 (3) 4	0 (0) 0
Skin and subcutaneous tissue disorders	1 (1) 1	0 (0) 0	2 (1) 2	1 (0) 1
**IP Dose Three: Number of participants received IP Dose Three**	**152**	**157**	**153**	**153**
**Any AEs**	**20 (13) 26**	**20 (13) 26**	**24 (16) 29**	**23 (15) 31**
Gastrointestinal disorders	3 (2) 4	3 (2) 3	1 (1) 1	4 (3) 5
General disorders and administration site conditions	1 (1) 1	2 (1) 2	2 (1) 2	2 (1) 2
Infections and infestations	13 (9) 16	13 (8) 14	18 (12) 21	16 (11) 19
Respiratory, thoracic and mediastinal disorders	3 (2) 3	3 (2) 4	4 (3) 4	2 (1) 2
Skin and subcutaneous tissue disorders	2 (1) 2	3 (2) 3	1 (1) 1	3 (2) 3
**IP Dose Four : Number of participants received IP Dose Four**	**150**	**155**	**149**	**151**

MedDRA System Organ Class	RV3-BB 1.0 × 10^7 FFU/mL (Neonatal) (N=170) n (%) m	RV3-BB 3.0 × 10^6 FFU/mL (Neonatal) (N=172) n (%) m	RV3-BB 1.0 × 10^6 FFU/mL (Neonatal) (N=169) n (%) m	RV3-BB 1.0 × 10^7 FFU/mL (Infant) (N=173) n (%) m
**Any AEs**	**20 (13) 27**	**22 (14) 27**	**21 (14) 26**	**13 (9) 15**
General disorders and administration site conditions	0 (0) 0	0 (0) 0	0 (0) 0	1 (1) 1
Infections and infestations	17 (11) 23	18 (12) 20	17 (11) 18	12 (8) 13
Respiratory, thoracic and mediastinal disorders	0 (0) 0	0 (0) 0	1 (1) 1	1 (1) 1
Skin and subcutaneous tissue disorders	1 (1) 1	1 (1) 1	4 (3) 4	0 (0) 0

Supplement Table 2: Fatal Serious Adverse Events

Fatal Serious Adverse Events (Preferred term)	RV3-BB $1x10^7$ FFU/mL (Neonatal) (N=170)	RV3-BB $3x10^6$ FFU/mL (Neonatal) (N=172)	RV3-BB $1x10^6$ FFU/mL (Neonatal) (N=169)	RV3-BB $1x10^7$ FFU/mL (Infant) (N=173)
**<28 days post any IP dose**				
Asphyxia	1	0	1	0
Sepsis	0	0	1	0
Septic shock	0	0	0	1
**Total**	**1**	**0**	**2**	**1**

Supplement Table 3: Severity of Episodes Rotavirus diarrhoea

	RV3-BB 1x10^7 FFU/mL (Neonatal) (N=178) n (%)	RV3-BB 3x10^6 FFU/mL (Neonatal) (N=179) n (%)	RV3-BB 1x10^6 FFU/mL (Neonatal) (N=175) n (%)	RV3-BB 1x10^7 FFU/mL (Infant) (N=179) n (%)	Total (N=711) n (%)
**Non Severe**	15 (8)	24 (13)	15 (9)	14 (8)	68 (10)
Rotavirus positive	2	2	1	1	6
Rotavirus negative	11	17	13	11	52
Missing (no sample)	2	5	1	2	10
**Severe (≥11)***	1 (1)	2 (1)	2 (1)	0 (0)	5 (1)
Rotavirus positive	0	1	2	0	3
Rotavirus negative	1	1	0	0	2
Missing (no sample)	0	0	0	0	0

*Episodes of diarrhoea were graded according to a modified Vesikari scoring system. Episodes of Diarrhoea with a modified Vesikari score of equal to or greater than 11 were defined as Severe (7).
